# Fundamental operations of the political in Beckett’s *Molloy*

**DOI:** 10.12688/openreseurope.19926.1

**Published:** 2025-05-23

**Authors:** Paul Stewart

**Affiliations:** 1 University of Nicosia School of Humanities Social Sciences and Law, Nicosia, Nicosia, Cyprus

**Keywords:** Beckett, Aristotle, Agamben, Bersani, Rancière, assimilation, political resistance

## Abstract

The political novel might be defined in terms of “genre,” or a novel’s overt intervention within a pre-established political field. However, this chapter contends that the process of personal individuation and incorporation within the State (or *polis*) is the fundamental operation of the political within the novel as a form. In order to sketch out the parallel, and paradoxical, operation of becoming an individual subject at the same time as, and in relation to, incorporation into a wider social state, this chapter examines how Samuel Beckett’s *Molloy* (1951) plots the resistance of its eponymous protagonist against both benign and coercive attempts to (a) define him as an individual, and (b) to assimilate him into the social body on that basis. Drawing on the works of Aristotle, Agamben, Bersani and Rancière, the chapter focuses on Molloy’s methods of avoidance of becoming a state-recognised and state-sanctioned subject and reads this avoidance as a form of resistance to the established *polity*. It is argued that Beckett’s non-relational art, of which *Molloy* is an early example, raises important theoretical issues concerning the interconnectivity of the political and the novel at a fundamental level. If the novel is dependent on just the sort of process that Molloy resists – that is on claims of individuality and relation –, can the novel as a form actively resist the political and resist assimilation and incorporation into a pre-established *polis*?

Until relatively recently, the works of Samuel Beckett have been little considered in light of the political, possibly, as Pascale Casanova contends, because Blanchot’s reading of Beckett’s novel *The Unnamable* ( *L’Innomable*) in the 1950s ( 11) led to an acceptance of Beckett as one who “lacked a past, an inheritance or a project” ( 12 on page 11). With greater awareness of archival sources, including the four volumes of Beckett letters, aspects of Beckett’s past and his inheritance have become clearer, allowing for a reassessment of his project in more political terms across a range of contexts. For example, Beckett’s relation to Ireland and the formation and ideology of the Irish Revival and of the Irish Free State has allowed many to see Beckett’s early prose as informed by, and a critique of, nationalism and nation building, or, indeed, as informed by a conflicted post-colonial sensibility. In *Beckett’s Political Imagination*, Emilie Morin has extended this to chart how the works intersect with, and very often challenge, political events and orthodoxies ranging from pre-War Nazi Germany (through which Beckett travelled extensively in 1937), post-War French recovery, and the Algerian War of Independence. As she notes, however, charting Beckett’s writing in political terms remains difficult “because his texts reimagine political history in ways that are idiosyncratic, juxtapose the factual and the symbolic, and play with allegory and historical anachronism” ( 17 on page 16).

Although there has been a movement towards the political in Beckett studies in recent years, one should note that the political possibilities of Beckett have been recognised and used by important post-War philosophers and theoreticians. Although Theodor Adorno once dismissed the thought of Beckett as a “political witness” ( 1 on page 125), he did elsewhere acknowledge that Beckett (along with Kafka) might gesture towards a new political art beyond the forms and restraints of “socialist realism, Sartrean commitment and Brechtian technique” ( 2 on page 89). Alain Badiou has frequently been drawn to Beckett as a writer who manages to escape the impasse of solipsism to create the conditions for happiness in relation to the Other, as, in Beckett’s *How It Is*, love “is neither fusion nor effusion. Rather it is the often painstaking condition required for the Two to exist as Two” ( 5 on page 28). For very different reasons, Gilles Deleuze has often turned to Beckett’s prose as a resource, particularly in what Foucault described as the project against “the fascism that causes us to love power, to desire the very thing that dominates and exploits us” ( 15 on page xiii).

These two trajectories – the academic and the broadly philosophical – mark a reassessment not only of what Beckett’s work might mean when looked at through a political prism, but, perhaps more importantly, how those works might disrupt, fragment and challenge political categorisations and aesthetic procedures. As this article argues, Beckett’s *Molloy* might offer a re-imagining, or even a refusal, of the political as it is currently formed and, in so doing, challenge the aesthetic authority and practices of the novel form.

*Molloy* consists of two first person narratives: that of Molloy as he journeys towards his mother, and that of Moran, a private detective who is sent by a mysterious agency to track down Molloy, for reasons that are unclear, even to Moran. Both men are writing their accounts and the questionable facility of writing – of bringing anything ‘to book’, as it were – forms a constant thread in both narratives and both narratives can be seen as failed quests: Molloy never reaches his mother, and Moran never tracks down Molloy, although he increasingly comes to resemble Molloy in the collapse of his social relations and identity. Whether through ignorance or temperament, Molloy repeatedly refuses to be identified or to enter into social relations. In the figure of Moran, one can see how a bourgeois identity – buttressed by home, possessions and socially-sanctioned positions – is dismantled, leaving Moran to wonder if, in his final decrepit state, he is not in fact more free than he was before.

Molloy is detained by the police for some transgression of “public order, public decency” ( 7 on page 17) when resting with his bicycle and crutches as he enters into an unnamed town. His interactions with the police are far from satisfactory. First, he cannot produce the ID that the officer demands:

Your papers! he cried. Ah my papers. Now the only papers I carry with me are bits of newspaper, to wipe myself, you understand, when I have a stool. Oh I don’t say I wipe myself every time I have a stool, no, but I like to be in a position to do so, if I have to. Nothing strange about that, it seems to me. In a panic I took this paper from my pocket and thrust it under his nose. ( 7 on page 17)

The threatening superior officer at the station, who interrogates Molloy, is, “rewarded for his pains by the discovery that I had no papers in the sense that word had a sense for him, nor any occupation, nor any domicile, that my surname escaped me for the moment and that I was on my way to mother, whose charity kept me dying” ( 7 on page 19). Despite breaking the law by not having any ID, Molloy is let go to resume his wanderings, leaving him to wonder: “Were they of the opinion that it was useless to prosecute me? To apply the letter of the law to a creature like me is not an easy matter. It can be done, but reason is against it” ( 7 on page 21). Molloy evades the law and police by being beneath their notice, he is a ‘creature’ without home, job, or even, perhaps, family. As he presents himself as not of the state, the state does not feel obliged to recognise him, even when recognition would be in the form of punishment.

Molloy’s refusal, or inability, to account for himself before the state in a manner that the state finds acceptable is one of many instances in his narrative through which he manages to remain separate from forms of social relation. This asocial nature did not escape the censure of Georg Lukács. In “The Ideology of Modernism”, Lukács famously included Beckett among the modernists whom he felt had abandoned both the form and the purpose of literature. The ‘“modernist” antirealism” ( 16 on page 1218), as exemplified by Joyce, Kafka and Beckett’s novel Molloy, reveals its ideology through the formal nature of its works, and it is an ideology based on the “ontological view governing the image of man [as] by nature solitary, asocial, unable to enter into relationships with other human beings” a characterisation of Molloy with which few would argue ( 16 on page 1219). For Lukács, such ontological solipsism negates the very basis of literature’s connection with the real world, and, as man is defined as an Aristotelian *zoon politikon*, is unable to answer the “basic question” of all literature “what is Man?” ( 16 on page 1219) In contrast, the individual of modernism is irredeemably alone, fragmented, and as “inexplicable to others as to himself” ( 16 on page 1222) as psychopathology replaces social personality. This “flight into psychopathology” might be a protest against the alienation, capitalisation and “catastrophe” of the modern world post-1914, but such a protest is futile as it “contains no concrete criticism”. If literature is to have an impact as protest – if it is to intervene in the political – then Lukács is very clear what it must include: “In any protest against particular social conditions, these conditions themselves must have the central place” ( 16 on page 1224). In Lukács’ terms, Beckett’s work is unable, and ideologically unwilling, to enter into a political relation with the world. As it might be said to abstract “particular social conditions” and stress the psychopathology of its protagonists, so Beckett’s work effectively negates the possibility of the literary intervention in the political and this amounts to “the negation of art” as such ( 16 on page 1232).

Beckett’s rejection of the social in Molloy can be seen through a variety of prisms; not least how violence is deployed and how this connects with the political. Beckett’s works are replete with moments of violence and one might be tempted to draw a distinction between acts of violence against representatives of the state and those acts of violence directed against the individual. When Mercier and Camier beat a policeman to death, one can easily see it as a violence directed towards not only the man, but also the political institutions for which the policeman acts ( 6 on page 75–77). But, as in the case of Molloy and a lonely charcoal burner, or Moran and a stranger in the woods whom he beats to a pulp, the link between the man accosted and the political is less easy to discern. It might appear to be an attack directed only at the man and, on that account, more a seemingly irrational irruption of violence. However, the acts against state representatives are no different in kind to acts against the individual as they are both a reaction against sociality itself and, therefore, against the very foundations of the political in the Aristotelian concept of the *polis*. Wilfully, and violently, Molloy and Moran exclude themselves from the political as it is currently framed.

A possible entry point is that of the social worker who offers Molloy an apparent kindness in the police station. In this room “full of dark forms, crowding in a dark place”, Molloy is initially ignored – “They paid no attention to me and I repaid the compliment” – until a “big fat woman dressed in black, or rather in mauve” rises up before him with the charitable offering of a cup of tea and slice of bread ( 7 on page 21). She, obviously, does pay attention to Molloy, who then offers the following advice:

Let me tell you this, when social workers offer you, free, gratis and for nothing, something to hinder you from swooning, which with them is an obsession, it is useless to recoil, they will pursue you to the ends of the earth, the vomitory in their hands. The Salvation Army is no better. Against the charitable gesture there is no defence, that I know of. You sink your head, you put out your hands all trembling and twined together and you say, Thank you, thank you lady, thank you kind lady. ( 7 on page 21)

Panic-stricken, he then flings the cup and saucer across the room. Here, swooning is a benefit which the charitable act “hinders”; the charity workers of the world, from whom Molloy would presumably wish to “recoil” in horror, “pursue” their charitable targets, as if police in pursuit of a miscreant. Finally, the defenceless target of the charitable gesture has no choice but to adopt an identifiable position of supplication and degradation when all he wanted was to remain as one dark form amongst other dark forms to whom no one paid attention.

Molloy is being identified here, and expected to act as if he were, one of the excluded of the world. The charitable act identifies Molloy as part of a group that needs charity; that needs their marginal status addressed if inclusion is to be successful. The Charity Commission of the United Kingdom has provided guidelines for charities wishing to foster social inclusion. Its terms, whilst laudable, perhaps suffer from a certain deafness towards implications for figures such as Molloy. Firstly, social exclusion is defined as:

the phenomenon where particular people have no recognition by, or voice or stake in, the society in which they live. The causes of social exclusion are multiple and usually appear connected with factors affecting a person’s or community’s social or economic circumstances, where the effect prevents people from participating fully in society ( 13).

There are a number of underlying assumptions here: firstly, that those who are marginalized wish to be recognised by the society in which they already live (Molloy does not want to be recognised and no alternative seems possible). Secondly, the desirability of being included is never in doubt as exclusion “prevents” people from social participation, thus making society the good from which exclusion is bad. This assumption that society is the good permeates throughout the charity commission’s guidelines on how to draft a charity’s charitable aims. They offer, amongst others, the following example: “To promote social inclusion for the public benefit by preventing people from becoming socially excluded, relieving the needs of those people who are socially excluded and assisting them to integrate into society” ( 13). Indeed, all the examples given in the guidelines underline the public benefit of the charitable act and this is a legal requirement in the UK whereby “every organisation set up for one or more charitable aims must be able to demonstrate that its aims are for the public benefit if it is to be recognised and registered as a charity in England and Wales” ( 13). If the charitable act is meant to be to help an individual, it can only achieve charitable status if a wider, social benefit accrues. Indeed, the need to prove public benefit suggests that society is the prior term to which those excluded must integrate, and on that basis one returns to Aristotle. For Aristotle, as Lukács emphasises, man is a political animal by nature, but the polis comes before the man: “the state has a natural priority over the household and any individual among us. For the whole must be prior to the part. It is clear then that the state is both natural and prior to the individual” ( 4 1253aI8) Only man’s relation to the state, his membership of it and his function within it, warrant his identification as a man. However, this assertion is tempered in the *Politics* by a distinction between “man” and the limits of man: “It follows that the state belongs to the class of objects which exist by nature, and that man is by nature a political animal. Any one who by his nature and not simply by ill-luck has no state is either too bad or too good, either subhuman or superhuman…” ( 4 1253a1) The superhuman is one who is entirely self-sufficient and therefore has no need of the support of the state and no need of the social. He appears as a god, beyond all man-made structures. The subhuman is illustrated by the animal and the slave, neither of which “participate in happiness, nor in a life that involves choice” ( 4 1280a21). If the state is arranged to achieve the “good life […] both communally and individually” ( 4 12378b15) then the slave who can have no access to happiness cannot be a member of the state by definition, nor can he or she participate in the defining characteristic of the member of the state: to “be able to rule and be ruled well” ( 4 1277a25). There is an apparent reciprocity to this notion of the good citizen as, although one is ruled over, one may also rule at some point, as if being subjugated gave access to sovereignty and power. Molloy, one should remember, styled himself as a “creature” rather than as a man when faced with the rule of law, effectively making himself unable to rule and be ruled well.

Molloy’s violent reaction to the social worker reveals that he has no desire to be included in the state and yet, in order for the public benefit of inclusion to occur, he must adopt an identifiable position as an excluded individual, as if his individuality were itself paradoxically dependent on him belonging to a group. It is against such a paradoxical inclusionary model that Molloy’s and Moran’s violence is directed.

In Molloy’s possible murder of the charcoal-burner what is precisely at stake is the possibility of entering into a social relation: the charcoal-burner offers Molloy shelter, begging him to share his hut. Moreover, from the perspective of society, this social relation would be between two people that appear to be excluded. They are literally removed from the state in that the crippled Molloy is lost in the woods and the charcoal-burner, who Molloy speculates must have been born in the woods, has no idea how to get to the nearest town. Like a dark Arden, the forest is a site of exclusion. If we imagine Molloy accepting the offer of shelter, then a little commune of the dispossessed on the margins of society would ensue. It is the very similarity between the two that is dangerous. It is, perhaps, for this reason that Molloy’s act of violence is an assertion not of impotence, but of potency:

People imagine, because you are old, poor, crippled, terrified, that you can’t stand up for yourself, and generally speaking that is so. But given favourable conditions, a feeble and awkward assailant, in your own class what, and a lonely place, and you have a good chance of showing what stuff you are made of. And it is doubtless in order to revive this possibility, too often forgotten, that I have delayed over an incident that is of no interest in itself, like all that has a moral. ( 7 on page 86)

Here, Molloy’s violence is an utter rejection of sameness based on marginalization. He and the charcoal-burner may be in the same “class”, but he does not accept this basis for some sort of social relation. Rather, his act of violent assertion rejects the prescribed role of the unfortunate in need of recuperation and integration. He asserts his difference, and does so with extreme, possibly murderous violence. 

Moran’s murder of yet another stranger in the woods shares many of the attributes already laid out with regard to the charcoal-burner. If Molloy, from the perspective of society, is one of the marginalized, then Moran is rapidly becoming one too; he is becoming physically denuded, his relationship with his son is breaking down, and he wanders in the woods not knowing how to achieve his goal. Once again, the similarity of the two figures of Moran and the stranger is emphasised:

The face which I regret to say vaguely resembled my own, less the refinement of course, same little abortive moustache, same little ferrety eyes, same paraphimosis of the nose, and a thin red mouth that looked as if it was raw from trying to shit its tongue. ( 7 on page 157–158)

As Molloy in his zone of dark forms in the police station, the stranger emerges from an inchoate space, a “dim man, dim of face and body, because of the dark” that gradually becomes individuated. In due course, Moran’s violence is activated by a rather ambiguous gesture from the stranger: He “thrust his hand at me” ( 7 on page 158). Although “thrust” suggests a certain aggression, one must remember that this is Moran’s characterisation and that what might be described is the extension of an open hand, even perhaps the offering of a handshake. No matter what the stranger’s intention, Moran’s reaction, whilst veiled within the text because he does not want to give way to literature, is extremely brutal. He pulps the man’s face with such force that only later does he come across a stray ear whilst looking for his keys, the loss of which is also an indication of Moran’s further estrangement from society. Crucially, this extreme violence means that the stranger “no longer resembled” Moran.

One can therefore discern that the Moran and Molloy violence is directed against a certain sameness upon which a nascent sense of identification and society might be built. In terms of the politics of inclusion, this is a rejection of even the most basic, and apparently innocent, form of assimilation on an interpersonal level. However, one might wish to question why murderous violence is necessary in this context.

The assertion of extreme violence by those marginalised, or becoming marginalised, might first be considered from the perspective of Agamben’s concept of the *homo sacer*, particularly in the manner in which the claim to violence might be seen to upset Agamben’s analysis of how sovereign power is initiated and maintained. Agamben sees the paradoxical figure of the *homo sacer* from archaic Roman law as the basis of state power inasmuch as this “bare life” is declared to be unfit for ritual sacrifice and yet anyone who kills the *homo sacer* will not be guilty of homicide. As Agamben explains:

3.2. This violence – the unsanctionable killing that, in his case, anyone may commit – is classifiable neither as sacrifice nor as homicide, neither as the execution of a condemnation to death nor as sacrilege. Subtracting itself from the sanctioned forms of both human and divine law, this violence opens a sphere of human action that is neither the sphere of *sacrum facere* nor that of profane action. ^1^

This sphere of action is what Agamben identifies as the “sovereign sphere” in which political power is legitimated, thus suggesting that this creation of “bare life” (i. e. he who may be killed but not sacrificed) is the “originary activity of sovereignty” (3.2) and that “life exposed to death (bare life or sacred life) is the originary political element” (4.2). This paradoxically marginal figure is excluded under the threat of murderous violence that, as the state claims that violence, will not be murder at all, and, as it cannot be sacrificed, the figure is also excluded from divine law. As Agamben emphasises, this amounts to the state founding its sovereignty on the assertion of who can be killed and who cannot and the creation of bare life that can be killed with impunity. Hence, as Agamben puts it, “the first foundation of political life is a life that may be killed, which is politicized through its very capacity to be killed” (4.1). So, the *homo sacer* – and Agamben points to those interned in concentration camps and to modern refugees – is the fulcrum upon which sovereignty depends:

His entire existence is reduced to a bare life stripped of every right by virtue of the fact that anyone can kill him without committing homicide; he can save himself only in perpetual flight or a foreign land. And yet he is in a continuous relationship with the power that banished him precisely insofar as he is at every instant exposed to an unconditioned threat of death. He is pure zoē, but his zoē is as such caught in the sovereign ban and must reckon with it at every moment, finding the best way to elude or deceive it. In this sense, no life, as exiles and bandits know well, is more “political” than his. (4.2)

This description sounds very much like a common method of viewing Beckett’s moribunds, Molloy and Moran amongst them: excluded, in perpetual flight and exile, yet harassed by the very forces of power which they would wish to avoid. In this context, Molloy’s and Moran’s acts of murderous violence take on an added significance. Rather than accepting the identity of a *homo sacer* figure, a defenceless, marginalized bare life against whom violent death is threatened, Molloy and Moran lay claim to violent action themselves. If they are political figures, they are so in that they refuse to accept the violent premises of exclusion and inclusion on which the political as it is currently framed depends, according to Agamben. They may be figures of “bare life”, but in their murderous violence, they assert that they will not be the mere victims of sovereignty’s originary forces; they will not take part in the creation or legitimation of a political realm.

One might consider, however, whether this refusal to be co-opted into a pre-established political realm could amount to a form of resistance upon which a new formulation of the political might be built. Arguably, this is what Leo Bersani envisaged when, in the closing paragraphs of *Homos*, he suggested that Beckett and Jean Genet “compel us, perhaps in spite of themselves, to re-think what we mean and what we expect from communication, and from community” ( 9 on page 181). Beckett’s name is added to Bersani’s study of what he terms the “gay outlaws” – Gide, Proust and Genet – in a rallying call to think a new form of the political; a form which is predicated on an initial recognition, and then maintenance, of the “unrelated subject” as this would be “the precondition for any viable reconstruction of [new] social relations. For such a reconstruction will have to take into account the persistence of unrelatedness, the priority of unrelatedness in the social itself” ( 10 on page 26–27). As I have argued elsewhere ( 19), this hoped for reconstruction is doubtful in the case of Beckett as it is dependent on the experience of jouissance that is “self-shattering” in so far as it “disrupts the ego’s coherence and dissolves its boundaries” which leads, paradoxically, to a form of “anti-identitarian identity” ( 9 on page 101).

Similarly, one might wish to use Molloy’s and Moran’s refusal to be identified through relation – be it in the terms the state sanctions, or in terms of interpersonal connections – as a means for a reconstruction of the political through Rancière’s concept of dissensus. Certainly, there seems to a great deal of congruence between Molloy’s and Moran’s trajectories and the operation of political consensus that Rancière identifies as the “aim […] to produce an identity between law and fact, such that the former becomes identical with the natural life of society. In other words, consensus consists in the reduction of democracy to the way of life or ethos of a society – the dwelling and lifestyle of a specific group” ( 18 on page 72). Before Moran sets off on his pursuit of Molloy, his life is very much one of a stable, specific group – a bourgeois homeowner, employee and parent – which adheres to the ethos of the society as a whole. This is precisely what he loses as he journeys, unsuccessfully, towards Molloy. As for Molloy, he might be seen as resisting the more coercive forms of consensus that “consists in the attempt to dismiss politics by expelling surplus subjects and replacing them with real partners, social and identity groups and so on ( 18 on page 71). As previously argued, Molloy refuses to be identified as a part of a distinct social group – those of need of charitable aid, for example – and embraces his expulsion from the social sphere.

Molloy’s and Moran’s rejection of a “bare life” as Agamben posits it and as argued above, might give further grounds for seeing them as compatible with “a capacity for staging scenes of dissensus” ( 18 on page 69) as Rancière explicitly hones his concept in distinction with Agamben. For Rancière, Agamben “de-populates the political stage” by

Incorporating the political exception into state power, posited as that which stands face to face with bare life. This opposition is then turned into a complementarity. The will to preserve the realm of pure politics ultimately has politics vanish in the pure relationship between state power and individual life. So politics gets equated with power and power itself gets increasingly construed as an overwhelming historico-ontological destiny from which only a God can save us. ( 18 on page 67)

In effect, then, Agamben is bound within a logic of relation, be it through opposition or complementarity. In contrast, Rancière sees the site of dissensus as one of supplement, or “the count of the uncounted – or the part of those who have no part” ( 18 on page 70). However, this supplement is configured as a communal position; “those who have no part” rather than “the individual who has no part”. As Rancière conceives it, dissensus might be “a division inserted in ‘common sense’: a dispute over what is given and about the frame within which we see something as given” ( 18 on page 69), yet the sense of the common must remain. Indeed, the collective is precisely necessary for the site of a new form of “dissensual ‘commonsense’” ( 18 on page 139). Binding Moran and Molloy to such a commonality would be to submit them once again to the logic and regime of relation when they vehemently and violently resist any identification upon which community might be built.

Despite the theoretical frames of the political which seem to have some degree of congruence with Molloy – from Aristotle to Agamben, Bersani to Rancière – all falter on the fundamental question of relation. There is no community, no common sense, without a prior relation. Furthermore, this question troubles the ontological premise of the novel itself. The concept of the reader coming into relation with the text – forming an imaginary community with the characters or narrator or the writing itself – is perhaps the premise upon which the novel as novel functions. If one were to repeatedly deny this relation, the novel would cease to function as a form of communication between one body and another, or, indeed, cease to be a case of the novelist apprehending and representing an exterior stimulus. Beckett was aware of precisely this problem as he argued for an art of non-relation in the *Three Dialogues with Georges Duthuit,* published in Transition in 1949. In the dialogues analysing the painters Tal Coat, Masson and Bram van Velde, Beckett charts a movement away from relational forms of art (the “farce of giving and receiving”) and “its attempts to escape from [a] sense of failure, by means of more authentic, more ample, less exclusive relations between representer and representee” ( 8 on page 125). For Beckett, this is to ignore the “increasing anxiety of the relation itself, as though shadowed more and more darkly by a sense of invalidity, of inadequacy, of existence at the expense of all it excludes” ( 8 on page 124). The case of van Velde is that, according to the Beckett figure in the Dialogues, he is the first to “submit wholly to the incoercible absence of relation” and to avoid the “possessiveness” of an art based on a subject-object relation. Yet, no matter how radical this concept of non-relational art might appear to be, Beckett knows that non-relation is threatened to become the basis of a further relation: “I know that all that is required now, in order to bring even this horrible matter to an acceptable conclusion, is to make of this submission, this admission, this fidelity to failure, a new occasion, a new term of relation” ( 8 on page 125). To maintain oneself within the realm of non-relation, Beckett admits, is to place oneself “in an unenviable situation, familiar to psychiatrists” ( 8 on page 126).

To apply this dynamic of the non-relational to the realm of the political, it is the wish to maintain the individual in a state of utter separation, despite the logical pull of making this separation the occasion for a new form of the political. One might wish to see non-relation as form of nascent community but Beckett’s characters refuse to fall into that “farce of giving and receiving” which can be seen as a power dynamic not unlike Aristotle’s defining characteristic of the member of the state as one who is “able to rule and be ruled well” ( 4 1277a25). This unenviable situation of being in non-relation might be “familiar to psychiatrists” and a “flight into psychopathology” but it is also a means of eluding the power structures and restrictions of the political and, as Terry Eagleton has pointed out of Beckett, perhaps it is “better to suffer the pains of self-dispossession than court the perils of dominion” ( 14 on page xxiv).

## Ethics and consent

Ethical approval and consent were not required.

## Data Availability

No data associated with this article.

## References

[1] Adorno TW Jones MT : Trying to understand endgame. *New German Critique.* 1982;26(1982):119–150. 10.2307/488027

[2] Adorno TW McDonagh F : On commitment. *Performing Arts Journal.* 1978;3(2):3–11. Reference Source

[3] Agamben G : Homo Sacer: Sovereign Power and Bare Life. Translated by Daniel Heller-Roazen. Stanford: Stanford University Press,1998. Reference Source

[4] Aristotle : The Politics. Translated by T. A. Sinclair and Trevor J. Sanders. London: Penguin,1998. Reference Source

[5] Badiou A : On Beckett. Edited by Nina Power and Alberto Toscana. Manchester: Clinamen,2003. Reference Source

[6] Beckett S : Mercier and Camier. London: Faber and Faber,2010. Reference Source

[7] Beckett S : Molloy. London: Faber and Faber,2009. Reference Source

[8] Beckett S : Proust and three dialogues with Georges Duthuit. London: John Calder,1987. Reference Source

[9] Bersani L : Homos. Cambridge MA.: Harvard University Press,1995. 10.4159/9780674020870

[10] Bersani L Dutoit U : Acts of impoverishment: Beckett, Rothko, Resnais. Cambridge, MA.: Harvard University Press,1993. Reference Source

[11] Blanchot M : Où maintenant? Qui maintenant?In: *La Nouvelle Revue Française.* 1953;10:678–686.

[12] Casanova P : Samuel Beckett: anatomy of a literary revolution. Translated by Gregory Elliott. London: Verso,2006.

[13] Charity Commission of the U.K.: The promotion of social inclusion. Accessed April 23, 2024. Reference Source

[14] Eagleton T : Foreword: nothing new. In: *Beckett and Nothing: Trying to Understand Beckett.*Edited by Daniela Caselli. Manchester: Manchester University Press,2010;xiv–xxvi.

[15] Foucault M : Preface. In: Gilles Deleuze and Félix Guattari, *Anti-Oedipus: Capitalism and Schizophrenia.*Translated by Robert Hurley, Mark Seem and Helen R. Jane. London: Penguin,2009;xi–xiv.

[16] Lukács G : The ideology of modernism. In: *The Critical Tradition: Classic Texts and Contemporary Trends.*Edited by David H. Richter. Boston: Bedford,1998;1218–1232.

[17] Morin E : Beckett’s political imagination. Cambridge: Cambridge University Press,2017. 10.1017/9781108284011

[18] Rancière J : Dissensus: on politics and aesthetics. Edited and translated by Steven Corcoran. London: Continuum,2010. Reference Source

[19] Stewart P : Sex and aesthetics in Samuel Beckett’s work. London: Palgrave Macmillan,2011. 10.1057/9780230339279

