# Supplementary material for: Fundamental operations of the political in Beckett’s Molloy
Source: Open Res Eur. 2025 May 23;5:141. [Version 1] doi: 10.12688/openreseurope.19926.1 (PMC12280865; doi:10.12688/openreseurope.19926.1)
Supplement: Peer review history

# Reviewer report: Reviewer response for version 1

**DOI:** 10.21956/openreseurope.21554.r55555

**Published:** 2025-07-26

**Authors:** Matteo Nicolini

**Affiliations:** 1 Verona University, Verona, Italy

In his *“Fundamental Operations of the Political in Beckett’s Molloy” , *Paul Stewart provides us readers with new insights as regards the political in Samuel Beckett. After reappraising the "reassessment of his project in more political terms across a range of contexts", the author leads us to a renovated (and convincing) reading of Beckett’s *Molloy.*

 I find the idea of re-imagining/refusal of the political extrememly compelling, particulalry beacuse these trajectories are assessed against "the aesthetic authority

 and practices of the novel form".

 The work is original in terms of both material and argument. As for the material, the novelty lies in the way Stewart interweaves Beckett's novel, literary criticism related to it, and the patterns drawn from Aristotle. The idea of probing Aristotle's *Politics* to assess the "paradoxical inclusionary model" challenged by both Molloy and Moran in terms of violence is decisively intriguing. Indeed, it supplies a fascinating reading of the relation between the state, the sameness that it produces within a given community, and the way novels might offer a counter-representation of political bonds. Likewise, Agamben's notion of bare life is fittingly criticised.

 Far from being the embedment of the *homines sacri*, both characters, as Stewart states, "will not take part in the creation or legitimation of a political realm", because they locate themselves outside traditional political taxonomies.   

 The arguments are persuasive and compelling, and the research article does contribute to the cultural, historical, social understanding of the field. The way he departs from Agamben's predicament as regards bare life is refreshing. Likewise, I appreciate the way Stewart reappraise the distruptive potential of Beckett's idea of "non-relation".

 I only suggest that the author arrange the text in sections, stating his main points in a clearer way, also turning the last paragraph into a concluding section. This will make his arguments even more compelling and cogent.

If any, are all the source data and materials underlying the results available?

Yes

Is the work original in terms of material and argument?

Yes

Is the work clearly and cogently presented?

Partly

Is the argument persuasive and supported by evidence?

Yes

Does the research article contribute to the cultural, historical, social understanding of the field?

Yes

Does it sufficiently engage with relevant methodologies and secondary literature on the topic?

Yes

Reviewer Expertise:

law and literature

I confirm that I have read this submission and believe that I have an appropriate level of expertise to confirm that it is of an acceptable scientific standard.

# Reviewer report: Reviewer response for version 1

**DOI:** 10.21956/openreseurope.21554.r55551

**Published:** 2025-07-21

**Authors:** A. Arun Daves

**Affiliations:** 1 Jawahar Science College, Neyveli, Tamil Nadu, India

Paul Stewart’s article offers a compelling and original reading of *Molloy* as a site of political resistance. Drawing on Aristotle, Agamben, Bersani, and Rancière, it argues that Beckett’s characters resist incorporation into the polis through non-relation and radical exclusion. The analysis is theoretically rich, well-structured, and supported by close textual readings. The abstract, methods, and references are all appropriate and clearly presented. The work contributes meaningfully to both Beckett studies and political theory. Language is clear and scholarly.

If any, are all the source data and materials underlying the results available?

Yes

Is the work original in terms of material and argument?

Yes

Is the work clearly and cogently presented?

Yes

Is the argument persuasive and supported by evidence?

Yes

Does the research article contribute to the cultural, historical, social understanding of the field?

Yes

Does it sufficiently engage with relevant methodologies and secondary literature on the topic?

Yes

Reviewer Expertise:

British Literature

I confirm that I have read this submission and believe that I have an appropriate level of expertise to confirm that it is of an acceptable scientific standard.

# Reviewer report: Reviewer response for version 1

**DOI:** 10.21956/openreseurope.21554.r55081

**Published:** 2025-06-18

**Authors:** Alan Ali Saeed

**Affiliations:** 1 University of Sulaimani, Sulaymaniyah, Kurdistan Region, Iraq

Paul Stewart’s article, *“Fundamental Operations of the Political in Beckett’s Molloy,”* is a multifaceted and intellectually rich examination of Beckett’s text through the prism of political ontology and poststructuralist critique. The piece skillfully interweaves theoretical understandings from Aristotle, Agamben, Bersani, and Rancière to debrief the latent structures of power, passivity, and ethical support embedded in Beckett’s narrative form and character construction. Rather than offering a conventional reading of *Molloy*, Stewart proposes a reframing of the political as a set of relational operations shaped by ambiguity, marginality, and withdrawal.

 The article’s strength lies in its capacity to bridge literary analysis with philosophical discourse, underscoring Beckett’s novel as a site of contestation where language and politics converge. The methodological approach as grounded in close textual analysis and rigorous theoretical framing, is sound and persuasive. The argument contributes significantly to the expanding field of Beckett studies and political literary criticism, advancing a reading that is both original and critically astute.

 All sources are properly cited, and the article makes a meaningful cultural and theoretical contribution. It may benefit from a clearer articulation of historical context to assist interdisciplinary readers, but overall, it stands as a well-argued and thought-provoking piece of scholarship.

If any, are all the source data and materials underlying the results available?

Yes

Is the work original in terms of material and argument?

Yes

Is the work clearly and cogently presented?

Yes

Is the argument persuasive and supported by evidence?

Yes

Does the research article contribute to the cultural, historical, social understanding of the field?

Yes

Does it sufficiently engage with relevant methodologies and secondary literature on the topic?

Yes

Reviewer Expertise:

My research focuses on Modern and Contemporary English Literature, with emphasis on stream-of-consciousness, subjectivity, and Kurdish narratives in English. I also explore interdisciplinary approaches involving film, graphic novels, and postcolonial critique.

I confirm that I have read this submission and believe that I have an appropriate level of expertise to confirm that it is of an acceptable scientific standard.
